# Image–mask–spurious triplets dataset for geometry-aware road extraction from UAV imagery

**DOI:** 10.1016/j.dib.2026.113142

**Published:** 2026-08-07

**Authors:** Carlos A. Aguirre López, John R. Ballesteros, John W. Branch Bedoya

**Affiliations:** Universidad Nacional de Colombia, Sede Medellín Calle 59A No. 63 - 20, Código Postal 050034 Medellín, Antioquia, Colombia

**Keywords:** Orthophoto segmentation, Boundary regularization, Vision transformer, SegFormer, Multi-model benchmark, Drone photogrammetry

## Abstract

This article presents a dataset of 3309 image–mask–prediction triplets derived from UAV orthophotography over six municipalities in Antioquia, Atlántico, and Chocó, Colombia, where predicted segmentation outputs exhibit degraded geometric quality such as noisy boundaries, fragmented regions, and angular distortions relative to the reference geometry. The dataset provides paired instances of geometrically regular ground-truth masks and degraded segmentation predictions, enabling the development of post-processing algorithms for boundary regularization and road vectorization. Non-overlapping 512 × 512-pixel RGB tiles were extracted at a stride of 512 pixels, retaining only tiles with a minimum road coverage of 10%. The dataset is partitioned into training (2084 samples; 63.0%), validation (232 samples; 7.0%), and test (993 samples; 30.0%) subsets. Each sample consists of three co-registered files: a raw UAV image tile, a binary ground-truth mask produced through manual digitization, and a predicted mask generated by the best-performing architecture from a five-model benchmark (SegFormer-B3). Connected-component analysis identified 3562 discrete road segment instances, and integrity validation confirmed zero-dimension mismatches across all 3309 pairs. The predicted masks exhibit boundary irregularities, width distortions, and angular deviations whose explicit pairing with ground-truth references enables quantitative evaluation of geometry-aware refinement methods.

Specifications Table

**Table:** 

Subject	Earth & Environmental Sciences
Specific subject area	Binary semantic segmentation of urban road surfaces from UAV imagery for topology-aware vector extraction
Type of data	Image (PNG tiles, RGB, 512 × 512 px). Masks are provided as single-channel binary images, where pixel values are 0 (background) and 255 (foreground).
Data collection	RGB drone imagery was captured over 6 Colombian municipalities (Antioquia, Atlántico, Chocó) with GSD of 2.14–12.38 cm/px. Seven orthomosaics (EPSG:32,618) were annotated by two operators using Global Mapper Pro v26.0.0 to create binary masks. Via a Python pipeline, 512×512 px tiles were extracted (stride 512). Tiles with >50% no-data regions were discarded, and a filter retained only those with >10% road coverage to ensure sufficient geometry for boundary regularization analysis.
Data source location	*Country: Colombia* City/Region: Antioquia, Atlántico, Chocó. Municipalities: Amagá, Barbosa, Barranquilla, Nuquí, San Pedro de los Milagros, Urrao.
Data accessibility	Repository name: Zenodo Data identification number: https://doi.org/10.5281/zenodo.19655229 Direct URL to data: https://zenodo.org/records/19,655,229?preview=1&token=eyJhbGciOiJIUzUxMiJ9.eyJpZCI6ImYxMmM5OWQ3LTY1NzEtNDhmZS1hNTgxLThiNDBlMWZlNmU2MyIsImRhdGEiOnt9LCJyYW5kb20iOiIyYzNlM2JkZDU0MGFlMjY4MWMzMDQzOWJiOTU0MTg5MSJ9.vyrKOovPvg6Vj6fFODoImBuA8zakJemqisPjKz8_mW9KGhrjnV3VAxBXxZDzKm9_cZxo5kem1AADqCFr2r79pg
Related research article	None

## 1 Value of the Data

The dataset comprises 3309 image–mask–prediction triplets from UAV imagery over six Colombian municipalities across three departments, providing a georeferenced, manually annotated resource for an underrepresented geographic domain in aerial road segmentation benchmarks [1,2].

• The triplet structure (raw tile, manual ground-truth mask, and SegFormerpredicted) provides paired instances of geometrically regular references and degraded boundaries, allowing the isolated quantification of geometric artifacts such as edge roughness, width variations, and fragmented segments without requiring prior model training.
• The foreground coverage distribution (mean FG ≈ 32.50%, FG/BG ratio ≈ 0.48) presents a moderately balanced class representation that reduces sensitivity to loss function selection [3], while retaining sufficient background context for boundary delineation.
• The included SegFormer-B3 baseline (IoU = 0.8740, Dice = 0.9272), selected from a five-architecture benchmark, serves as a validation baseline for evaluating boundary-refinement frameworks [4].
• The multi-altitude acquisition across six municipalities (GSD range: 2.14–12.38 cm/px) introduces scale and geographic variability, enabling evaluation of model robustness to spatial resolution changes [5,6].
• The paired triplet structure enables direct benchmarking of boundary-refinement algorithms, polygon regularization methods, and raster-to-vector pipelines under controlled conditions.

## 2 Background

Road network extraction from aerial imagery is fundamental to geospatial analysis, urban planning, and infrastructure monitoring [1]. UAVs provide high-resolution, georeferenced imagery over complex urban environments [2], and the growing diversity of UAV datasets reflects increasing demand for domain-specific annotated resources [1].

Encoder–decoder architectures such as U-Net [7] have established baselines for binary road segmentation, while specialized variants incorporating spatial aggregation [8], frequency-domain awareness [2], directional priors [9] improve connectivity and boundary precision. Despite these advances, raster-based predictions remain prone to degraded outputs including noisy boundaries, disconnected regions, fragmented segments, width distortions, and angular deviations from the reference geometry [9].

These artifacts obstruct direct vectorization for GIS applications. Topology-preserving methods such as Skea-Topo [10] and TopoRF-Net [6], partially address this problem; however, no publicly available dataset that pairs ground-truth masks with curated model predictions exhibiting such geometric failure modes [1,5] exists. This dataset addresses that gap through image–mask–prediction triplets acquired over Colombian urban areas, a context underrepresented in public benchmarks [1]. Beyond extending geographic coverage, the dataset's scientific value lies in three research directions: (i) benchmarking boundary-regularization and polygon-simplification algorithms under controlled geometric degradation; (ii) supporting raster-to-vector road-extraction pipelines for GIS integration; and (iii) enabling transferability studies across UAV acquisition altitudes and ground sampling distances.

## 3 Data Description

The dataset is organized as a three-level directory hierarchy. Each split contains three subdirectories with spatially aligned files sharing identical filename stems:

**Table:** 

Path	Description
dataset/	Root directory
train/	Training partition — 2084 triplets
images/	RGB tiles (512 × 512 px, PNG)
masks/	Ground-truth binary masks (PNG, 8-bit grayscale)
predicted_masks/	SegFormer-B3 predictions — may contain segmentation errors (PNG)
val/	Validation partition — 232 triplets
images/	RGB tiles (512 × 512 px, PNG)
masks/	Ground-truth binary masks
predicted_masks/	SegFormer-B3 predicted masks
test/	Test partition — 993 triplets
images/	RGB tiles (512 × 512 px, PNG)
masks/	Ground-truth binary masks
predicted_masks/	SegFormer-B3 predicted masks

A supplementary metadata file (tiles_metadata.csv) accompanies the dataset, recording for each tile its unique identifier, source orthomosaic (municipality), pixel coordinates within the source raster, tile dimensions, foreground coverage ratio, affine transform, coordinate reference system (CRS), and assigned split. This file enables spatial traceability of every tile back to its origin orthomosaic.

**Fig. 1:**
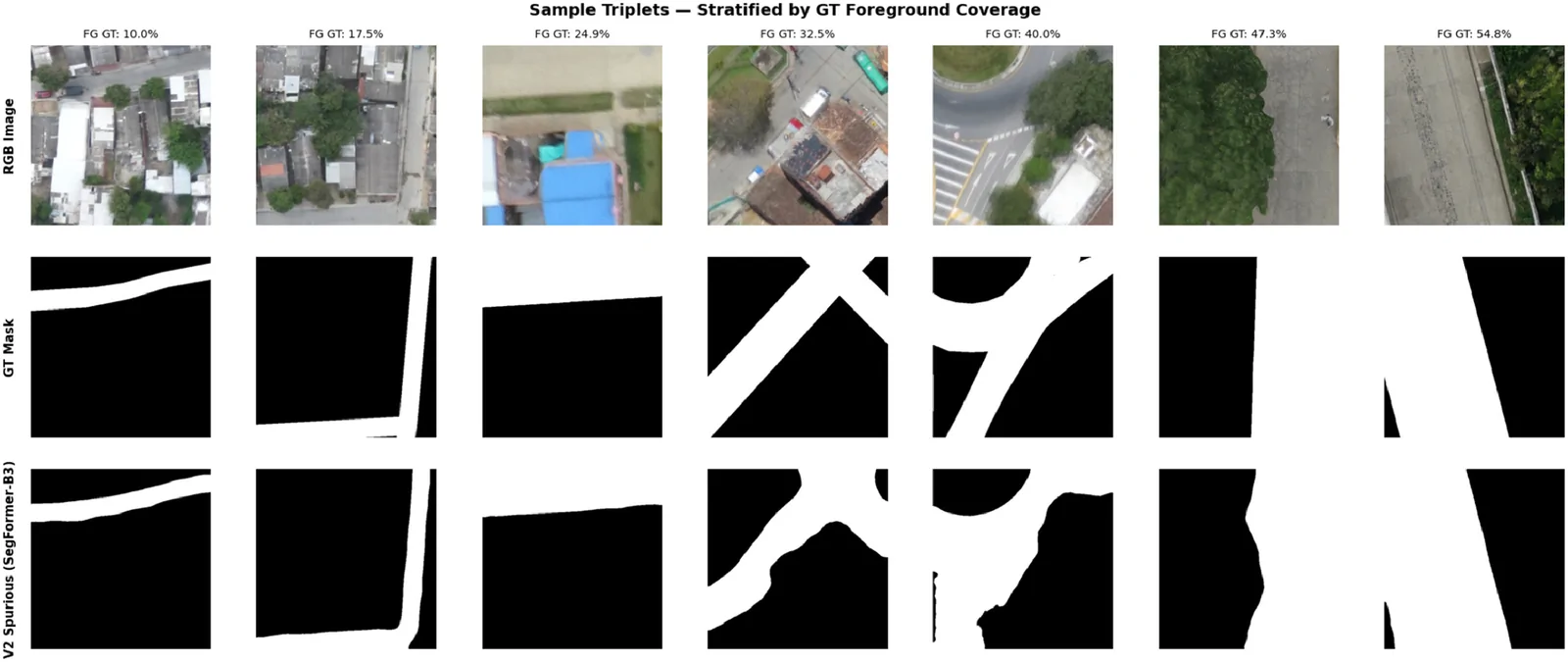
Representative image–mask–prediction triplets across the foreground coverage range (GT FG% from 10% to 54%). Top row: UAV image tile. Middle row: ground-truth binary mask. Bottom row: SegFormer-B3predicted mask. Note the geometric regularity of ground-truth boundaries versus segmentation errors (irregular contours, fragmented regions, angular deviations) present in predictions.

All files follow a consistent naming scheme based on a shared stem identifier (e.g., tile_00001.png), enabling deterministic programmatic loading without auxiliary index files. Each image tile in images/ has a uniquely corresponding ground-truth mask in masks/ and a predicted mask in predicted_masks/ with the same filename. Both ground-truth and predicted binary masks use a standard two-value encoding: road pixels are assigned intensity 255 (white) and non-road pixels 0 (black), consistent with sigmoid-thresholded binary segmentation output conventions (Fig. 1).

### 3.1 Spatial coverage per municipality

The number of tiles generated per orthomosaic of origin and the corresponding ground sampling distance are presented in Table 1.

**Table 1:** Spatial coverage and tile count per source orthomosaic.

Municipality	Department	Orthomosaic dimensions (px)	GSD (cm/px)	Tiles	% Total
Amagá	Antioquia	7979 × 10465	12.38	62	1.9%
Barbosa	Antioquia	12972 × 10864	6.81	212	6.4%
Barranquilla	Atlántico	13973 × 15082	9.21	435	13.1%
Nuquí (ortho 1)	Chocó	6066 × 6083	6.51	31	0.9%
Nuquí (ortho 2)	Chocó	7344 × 7193	6.51	39	1.2%
San Pedro de los Milagros	Antioquia	35768 × 38769	3.00	833	25.2%
Urrao	Antioquia	51313 × 87919	2.14	1697	51.3%
**Total**	**3 departments**	**7 orthomosaics**	**2.14–12.38**	**3309**	**100%**

All orthomosaics are projected in UTM Zone 18 N (EPSG:32,618). Tile counts reflect content-filtered totals (foreground coverage ≥ 10%).

### 3.2 Dataset statistics

The distribution and foreground coverage statistics across all three splits are presented in Table 2.

**Table 2:** Dataset split statistics and per-image foreground coverage.

Split	Samples	% Total	Mean FG%	Std FG%	FG/BG ratio
Training	2084	63.0%	32.35%	17.47%	≈ 0.48
Validation	232	7.0%	32.09%	18.75%	≈ 0.47
Test	993	30.0%	32.91%	18.18%	≈ 0.49
**Total**	**3309**	**100%**	**32.50%**	**17.77%**	**≈ 0.48**

Tiles were extracted without spatial overlap (stride = tile size = 512 px) and partitioned via stratified random sampling by source orthomosaic. A content-based filter excluded tiles with <10% foreground coverage to ensure sufficient road geometry for boundary regularization analysis.

**Fig. 2:**
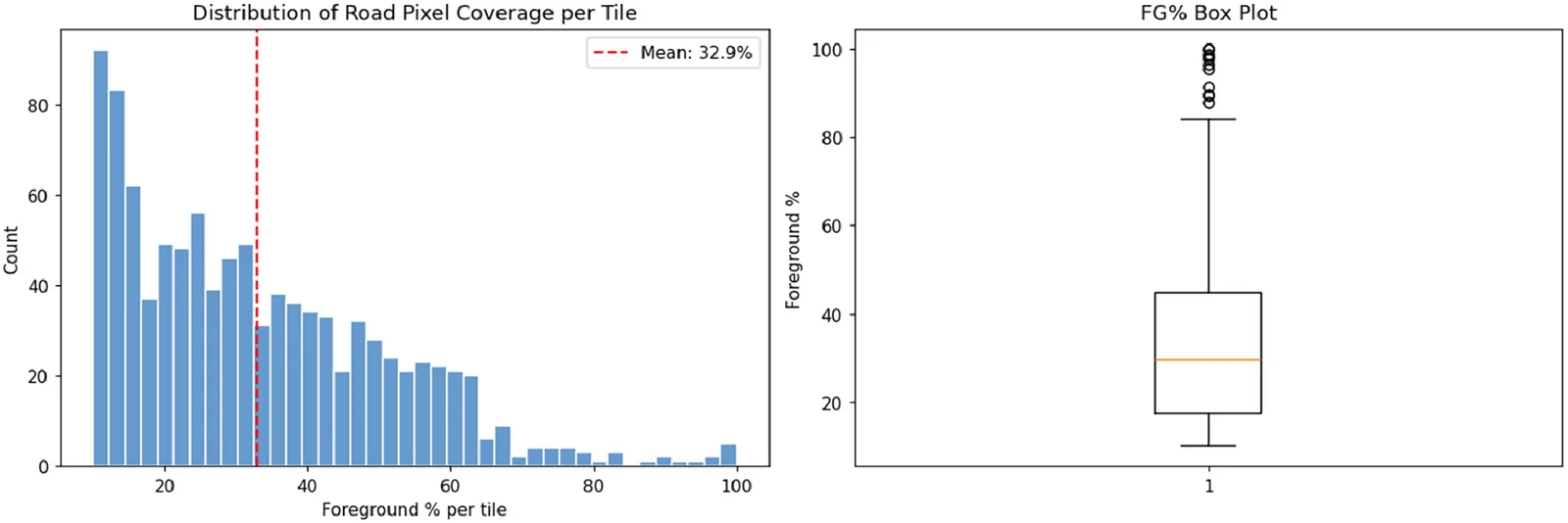
Distribution of foreground coverage (road pixel percentage) per tile across the full dataset (n = 3309). Left: histogram with mean at 32.9%. Right: box plot showing median at 29%, IQR from 18% to 44%, and outliers approaching 100%.

The distributional properties are corroborated by Fig. 2. The histogram (left) shows a positively skewed distribution with a mode at 15–20% foreground coverage, while the higher mean (32.5%) relative to the median (29%) confirms the influence of high-density tiles. The box plot (right) indicates an interquartile range of 18–44%, with values above 80% identified as outliers associated with complex road structures.

Overall, Fig. 2 demonstrates that the dataset captures a representative range of urban road densities, and the enforced 10% lower bound ensures sufficient geometric content for meaningful analysis.

### 3.3 Object instance count and mask integrity

Connected-component analysis (8-connectivity, scipy.ndimage.label) identified 3,562 discrete road segments across the 3309 tiles (mean: 1.08 segments per tile), exceeding the minimum of 50 unique object instances required by the Data in Brief image dataset guidelines. The non-overlapping tiling strategy (stride = tile size) ensures each segment is counted without spatial redundancy. Automated integrity checks confirmed that all 3309 masks are strictly binary ({0, 255}), with zero-dimension mismatches across all triplets; 3307 tiles contain both classes, while two all-white tiles represent valid samples where road pavement fills the entire tile extent; these tiles may be excluded from boundary-specific analyses as they lack an internal road/background boundary. The triplet structure facilitates the computation of geometric regularity metrics such as edge smoothness and boundary regularity, shape distortion, and topology connectivity by providing paired ideal and degraded samples for each road segment.

## 4 Experimental Design, Materials and Methods

### 4.1 Data acquisition

Aerial imagery was acquired using DJI Mavic 2 Pro equipped with a Hasselblad L1D-20c camera (1-inch CMOS sensor, 20 MP effective pixels, RGB). UAV surveys were planned and conducted by the authors (C. López and J. Ballesteros); all imagery is owned by the authors and licensed under CC BY 4.0. Flight missions were planned with 75% frontal overlap and 60% lateral overlap. Orthomosaic georeferencing relied exclusively on the UAV's onboard GNSS receiver; no ground control points (GCPs) or RTK/PPK correction were used, so absolute positional accuracy is bounded by the consumer-grade GNSS module of the platform. The platform was flown over six municipalities in Colombia: Amagá, Barbosa, San Pedro de los Milagros, and Urrao in the department of Antioquia; Barranquilla in the department of Atlántico; and Nuquí in the department of Chocó. Seven orthomosaics were generated across the six municipalities (two orthomosaics for Nuquí) using standard photogrammetric workflows, all projected in UTM Zone 18N (EPSG:32618). Variable flight altitudes across municipalities yield ground sampling distances (GSD) ranging from 2.14 cm/px (Urrao) to 12.38 cm/px (Amagá), resulting in spatial scale variability across the dataset [8,2].

### 4.2 Manual annotation protocol

Ground-truth binary masks were produced through a structured three-stage pipeline. First, road centerline vectors were obtained from OpenStreetMap (OSM) [11] as an initial geographic reference and manually adjusted to align with each orthophoto. Second, an optimum buffer distance was computed for each centerline to generate primitive mask geometries [11], and road boundaries were manually refined over each orthomosaic using Global Mapper Pro (v26.0.0). Annotation tasks were distributed by two annotators with experience in road digitization from aerial imagery; cross-validation was performed through mutual visual inspection of each other's digitized regions, with boundary discrepancies resolved iteratively until consensus was reached. Third, validated vector annotations were rasterized to produce single-channel binary PNG masks (road = 255, background = 0).

### 4.3 Image tiling pipeline

Orthomosaics and corresponding masks were subdivided into 512 × 512 pixel tiles using a sliding window with stride 512 pixels (zero overlap), ensuring each pixel appears in exactly one tile thereby avoiding pixel duplication between samples. Note that spatial autocorrelation may persist between neighboring tiles from the same orthomosaic [12]. Two sequential filters were applied: (i) a no-data filter removed tiles with >50% black pixels at orthomosaic borders (8699 tiles discarded), and (ii) a content-based filter excluded tiles with <10% foreground coverage (15,780 tiles discarded), retaining 3309 tiles. The content filter reflects the dataset's purpose: supporting geometry-aware penalization research requires sufficient road geometry (edges, junctions, angles) in each tile for meaningful boundary regularization analysis.

Image–mask pairs were extracted simultaneously to ensure pixel-perfect correspondence. Tiles were partitioned into training (2084; 63.0%), validation (232; 7.0%), and test (993; 30.0%) subsets via stratified random sampling by source orthomosaic, preserving per-municipality distribution andpreserving the proportional contribution of each source area to each partition. Because tiles from the same orthomosaic appear in all three subsets, this constitutes a tile-level split rather than a geographic-level split; therefore, test-set performance may overestimate generalization to unseen geographic areas. After partitioning, predicted masks were generated for all 3309 tiles using the best-performing segmentation model, SegFormer-B3 (Section 4) and stored in a dedicated predicted_masks/ subdirectory within each split, completing the image–mask–prediction triplet structure while preserving filename correspondence across all three components. For the training partition, these predictions are in-sample outputs (the model was trained on those images); consequently, training-split predictions may exhibit lower error rates than validation or test predictions. Users training downstream refinement models should consider cross-validation or out-of-fold prediction strategies if in-sample bias is a concern for their application.

### 4.4 Segmentation model benchmark

To select the architecture for predicted mask generation, five segmentation models were trained and evaluated: SegFormer-B3 [13], d-LinkNet [14], U-Net++ [15], DeepLabV3+ [16], and TransUNet [17]. All models were trained under identical conditions to ensure fair comparison.Training was performed on the 2084-sample training partition for up to 80 epochs (batch size = 2, gradient accumulation ×4, effective batch size 8) using a combined loss function of Binary Cross-Entropy and Dice Loss with equal weighting [3]:$L_{total}=0.5\cdot L_{BCE}+0.5\cdot L_{Dice},\;\text{where}\;L_{Dice}=1-\frac{2\sum y_{i}\cdot {\overset{ˆ}{y}}_{i}+\varepsilon }{\sum y_{i}+\sum {\overset{ˆ}{y}}_{i}+\varepsilon },\;\text{with}\;\varepsilon =1\times 10^{-7}.$

**Fig. 3:**
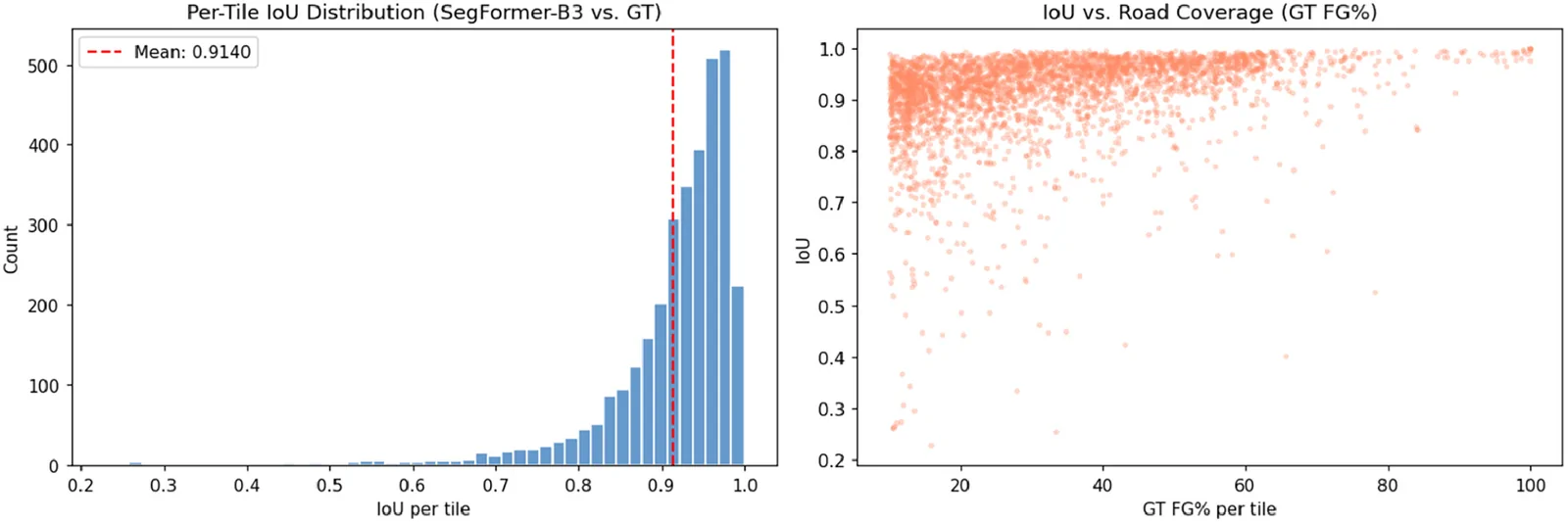
Per-tile IoU distribution of SegFormer-B3 predictions versus ground-truth masks on the full dataset. Left: histogram (mean IoU = 0.9140). Right: IoU versus GT foreground coverage, illustrating performance variability across road densities.

The AdamW optimizer [18] was used with a learning rate of 6 × 10⁻⁵ and weight decay of 1 × 10⁻⁴. A cosine annealing learning rate schedule [19] was applied to smoothly reduce the learning rate over the training horizon. Automatic mixed precision (AMP) was enabled to reduce memory consumption and accelerate training. Data augmentation consisted of random horizontal flips, vertical flips, and 90°-multiple rotations. The best checkpoint was selected by maximum validation IoU via early stopping (patience = 12 epochs). SegFormer-B3 achieved the highest test IoU (0.8740) and was selected to generate the predicted masks included in this dataset. Test performance for all five models is reported in Table 3. Predictions were binarized with a threshold of 0.5 (Fig. 3).

**Table 3:** Segmentation model benchmark - test set performance.

Model	IoU	Dice/F1	Precision	Recall
SegFormer-B3	0.8740	0.9272	0.9233	0.9436
D-LinkNet	0.8725	0.9300	0.9268	0.9365
U-Net++	0.8702	0.9284	0.9258	0.9350
DeepLabV3+	0.8380	0.9084	0.9149	0.9092
TransUNet	0.8197	0.8970	0.8984	0.9020

SegFormer-B3 was selected as the best-performing model based on the highest test IoU. All models were trained with identical data augmentation and early stopping on validation IoU.

### 4.5 Quality assurance

An automated Python pipeline (OpenCV 4.8.1, NumPy 1.26.4, SciPy 1.14.1) performed five sequential checks: (i) filename-stem correspondence across all three subdirectories (images, masks, predicted_masks) in each split, confirming 3309 complete triplets; (ii) dimensional uniformity (512 × 512 px for all triplets); (iii) binary mask integrity (values restricted to {0, 255}); (iv) per-tile foreground coverage profiling; and (v) connected-component analysis (8-connectivity) to quantify unique road segment instances (3562 total instances across the dataset). All checks passed without error.

### 4.6 Software and tools

The pipeline was implemented in Python 3.10.12 using OpenCV 4.x, NumPy 1.26.4, SciPy 1.14.1, and Rasterio 1.3.10 for image processing and geospatial I/O; PyTorch 2.4.1 for model training and inference; scikit-learn 1.5.2 for stratified dataset splitting; and Matplotlib 3.9.2 / Seaborn 0.13.2 for EDA visualizations. Road boundaries were digitized using Global Mapper Pro v26.0.0.

## Limitations

The dataset presents the following:

• Variable ground sampling distance (2.14–12.38 cm/px) across municipalities; fixed-GSD applications require spatial normalization [12].
• Positional accuracy: orthomosaic georeferencing relied solely on the UAV's onboard GNSS receiver (no GCPs or RTK/PPK correction), which may introduce centimeter-to-meter-level absolute positional error; relative geometric measurements within each orthomosaic are unaffected.
• Content-based filtering: tiles below 10% foreground coverage were excluded, removing sparse road segments unsuitable for presence/absence classification task.
• Limited geographic scope: all sites are in Colombia, and, Urrao contributes 51.3% of tiles, which may limit generalization to other regions [1].
• Multi-model benchmark: the predicted masks were generated by SegFormer-B3 (highest test IoU = 0.8740 among five evaluated architectures). The geometric artifacts observed may not cover all failure modes; researchers may generate predictions with additional architectures using the provided image–mask pairs.
• Residual annotation artifacts: inter-annotator agreement was not quantified with a formal metric, and photogrammetric stitching may propagate distortions to individual tiles.
• Tile-level splitting: tiles from the same orthomosaic appear across subsets; researchers requiring cross-site evaluation should re-partition at the orthomosaic level using tiles_metadata.csv.

To support full reproducibility, the repository includes the preprocessing scripts (tiling, content filtering, and stratified train/validation/test partitioning) and the training scripts for all five benchmarked architectures, together with the metadata file (tiles_metadata.csv) and the per-model test-set metric files used to generate Table 3. All code is available at the Zenodo repository associated with this dataset [20].

## Ethics Statement

The authors have read and follow the ethical requirements for publication in Data in Brief. The current work does not involve human subjects, animal experiments, or any data collected from social media platforms. All UAV flight operations were conducted in compliance with applicable Colombian civil aviation regulations.

## Declaration of Generative AI and AI-Assisted Technologies in the Manuscript Preparation Process

During the preparation of this work the author(s) used Claude (Anthropic) in order to assist with academic writing, structural organization, and language editing of manuscript sections. After using this tool/service, the author(s) reviewed and edited the content as needed and take(s) full responsibility for the content of the published article.

## CRediT authorship contribution statement

**Carlos A. Aguirre López:** Conceptualization, Data curation, Formal analysis, Methodology, Software, Visualization, Writing – original draft, Project administration. **John R. Ballesteros:** Supervision, Investigation, Resources, Validation, Conceptualization, Methodology, Writing – review & editing. **John W. Branch Bedoya:** Supervision, Investigation, Validation, Conceptualization, Writing – review & editing.

## Data Availability

ZenodoImage–Mask–Spurious Triplets Dataset (Original data).

## Appendix Supplementary Materials

Image, application 1
